# The genome sequence of the Alpine Zephyr Blue, *Kretania trappi* (Verity, 1927) (Lepidoptera: Lycaenidae)

**DOI:** 10.12688/wellcomeopenres.24703.1

**Published:** 2025-08-11

**Authors:** Yannick Chittaro, Kay Lucek, Charlotte J. Wright, Joana I. Meier, Mark L. Blaxter, Wellcome Sanger Institute Tree of Life Management, Samples and Laboratory team, Wellcome Sanger Institute Scientific Operations: Sequencing Operations, Wellcome Sanger Institute Tree of Life Core Informatics team, Tree of Life Core Informatics collective, Project Psyche Community

**Affiliations:** 1 Info fauna, Neuchâtel, Switzerland; 2 University of Neuchâtel, Neuchâtel, Switzerland; 3 Tree of Life, Wellcome Sanger Institute, Hinxton, England, UK

**Keywords:** Kretania trappi, Alpine Zephyr Blue, genome sequence, chromosomal, Lepidoptera

## Abstract

We present a genome assembly from a female specimen of *Kretania trappi* (Alpine Zephyr Blue; Arthropoda; Insecta; Lepidoptera; Lycaenidae). The assembly contains two haplotypes with total lengths of 683.81 megabases and 597.58 megabases. Most of haplotype 1 (98.36%) is scaffolded into 20 chromosomal pseudomolecules, including the W and Z sex chromosomes. Haplotype 2 was assembled to scaffold level. The mitochondrial genome has also been assembled, with a length of 15.35 kilobases.

## Species taxonomy

Eukaryota; Opisthokonta; Metazoa; Eumetazoa; Bilateria; Protostomia; Ecdysozoa; Panarthropoda; Arthropoda; Mandibulata; Pancrustacea; Hexapoda; Insecta; Dicondylia; Pterygota; Neoptera; Endopterygota; Amphiesmenoptera; Lepidoptera; Glossata; Neolepidoptera; Heteroneura; Ditrysia; Obtectomera; Papilionoidea; Lycaenidae; Polyommatinae; *Kretania*; *Kretania trappi* (Verity, 1927) (NCBI:txid2505780)

## Background

The Alpine Zephyr Blue is a member of the Lycaenidae family which, following the molecular work of Talavera *et al*. (2013), has been placed in the genus *Kretania*. Within this genus, the status of the *pylaon* species complex has not yet been satisfactorily resolved, and opinions differ on the taxonomic level of various taxa (see for example Jutzeler & Lafranchis, 2020; Kudrna *et al.*, 2011; Kudrna *et al.*, 2015; Tshikolovets, 2011), including the Alpine endemic taxon *trappi* (Verity, 1927). The latter was formerly known as *Lycaena lycidas* (Trapp, 1863), which is now an invalid homonym. While some authors consider this taxon to be a subspecies (e.g. Kudrna *et al.*, 2015), and others a ‘semispecies’ (e.g. Lafranchis *et al.*, 2015), it is currently mostly accepted as a distinct species, including on the most recent European checklist ( Wiemers *et al.*, 2018).

The global distribution of *Kretania trappi* is limited to a few isolated populations in northern Italy – Val Venosta (see Huemer, 2004), and Aosta Valley ( GBIF Secretariat, 2023) – and Valais, southern Switzerland ( GBIF Secretariat, 2023). Although very localised, the species can be locally abundant. A few additional records exist in northern Italy (Val d’Antrona and Val Vigezzo according to Dufay, 1974) and in France (Vanoise) on the French-Italian border ( Pierre & Pierre, 1993), but there appear to be no established populations at these sites.

As with most lycenids, *Kretania trappi* shows considerable sexual dimorphism, with males having a blue-violet colouration on the upper wings, while females are brown. The white submarginal arrows on the underside of the hindwings are very broad or united in a well-defined white shade between the black postdiscal spots and the orange submarginal lunules ( Bálint & Kertész, 1990). Within the genus *Kretania* it is often difficult to distinguish taxa. For example, there is little difference in external morphology between *K. trappi* from the Alps and *K. sephirus* (Frivaldsky, 1835) from the Balkans, Rumania and Hungary, which also use the same host plant ( Fiedler *et al.*, 1992). However, the two species differ in male genital morphology ( Bálint & Kertész, 1990). *Kretania trappi* differs from species of the genus *Plebeius* in the absence of metallic blue spots on the margin of the underside of the hindwings, whereas certain *Polyommatus* species with similar appearance – *P. escheri* (Hübner, 1823) and *P. thersites* (Cantener, 1835) – have fewer long white spots between the black postdiscal spots and the orange submarginal lunules.

*Kretania trappi* is univoltine and imagos can be seen from late May to mid-August, depending on altitude. As a xerothermophile, the butterfly is found at altitudes between 800 and 2 000 m ( Ligue Suisse pour la Protection de la Nature, 1987), on rocky steppe slopes and in open thermophilous pine forests, where its host plant, the Stemless milk vetch ( *Astragalus exscapus*), grows in abundance. In the Alps, the distribution of the butterfly overlaps with that of its host plant, which is also restricted to the three regions mentioned above ( Becker, 2013). The use of Central Alps milk vetch ( *Astragalus alopecurus*) has occasionally been reported in northern Italy ( Ligue Suisse pour la Protection de la Nature, 1987).

The female lays one egg per leaf on the leaf’s underside on the host plant. The caterpillar hatches in summer and feeds until an intermediate larval stage before hibernating. It continues to grow in spring by feeding on fresh shoots of its host plant close to the ground ( Ligue Suisse pour la Protection de la Nature, 1987). Being at least facultatively myrmecophilous, the caterpillars are usually accompanied by ants. Lafranchis *et al.* (2015) indicate *Formica lugubris* and *F. lemani*, but given the long list of potential host ant species suggested by Fiedler (2021) for the *pylaon* complex, which included *trappi*, it is likely that many other ant species are involved. The caterpillar pupates in the upper chambers of small anthills ( Ligue Suisse pour la Protection de la Nature, 1987), usually located directly in within the host plant *Astragalus exscapus*.

With a very limited and fragmented distribution, linked to a plant that is itself localised and sometimes in need of targeted conservation measures ( Kienberg & Becker, 2017), *Kretania trappi* has been classified as vulnerable in Switzerland ( Wermeille *et al.*, 2014) and in Italy ( Balletto *et al.*, 2015). Largely associated with traditional pastoral activities, its habitats are sensitive to abandonment and overgrowth, while irrigation and intensification of agricultural practices (especially overgrazing, as cattle are fond of *Astragalus exscapus*) also threaten certain populations ( Wermeille *et al.*, 2014).

The reference genome of *Kretania trappi* will help to clarify the existing confusion regarding the taxonomic status of many taxonomic entities belonging to the genus *Kretania* also in the context of chromosome numbers ( Coutsis & De Prins, 2006). Within the *trappi* taxon itself, the reference genome allows future studies to resolve potentially cryptic diversity as the Val Venosta populations are sometimes attributed to a specific subspecies ( *delattini* according to Junge, 1971), while the populations of the Aosta Valley are attributed to the *augustanus* taxon by van Mentzner (1956). As demonstrated by DNA barcoding, the Val Venosta populations seem to be well differentiated from the other populations ( Dapporto *et al.*, 2022).

## Methods

### Sample acquisition

**Figure 1.**
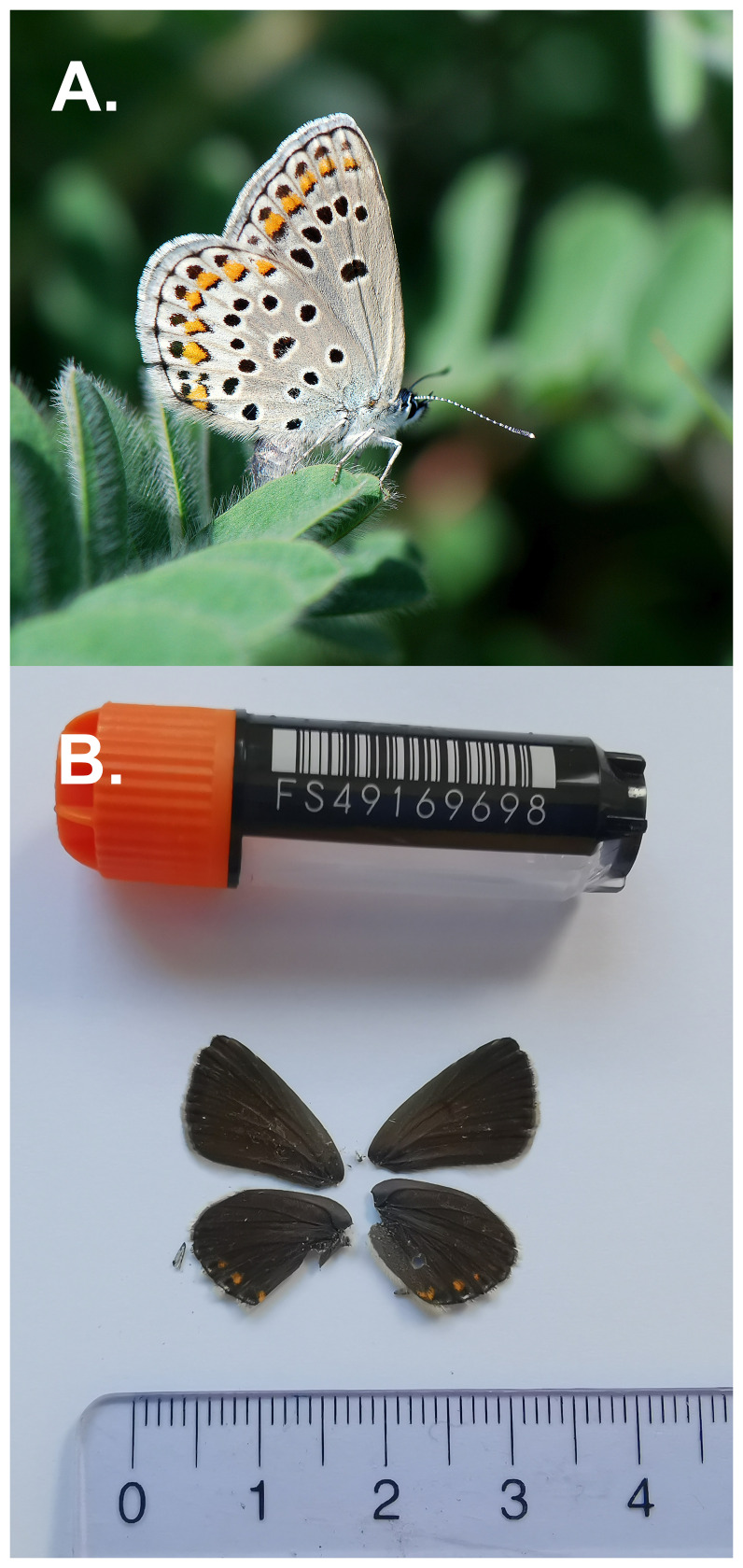
**A**. Live specimen of *Kretania trappi*; **B**. Voucher image of the *Kretania trappi* (ilKreTrap1) specimen used for genome sequencing.

The specimen used for genome sequencing was an adult female *Kretania trappi* (specimen ID SAN28000118, ToLID ilKreTrap1; Figure 1), collected from Simplon, Valais, Switzerland (latitude 46.2506, longitude 8.0273; elevation 2 000 m) on 01/06/2023. The specimen was collected and identified by Yannick Chittaro (Info Fauna, Neuchâtel, Switzerland).

### Nucleic acid extraction

Protocols for high molecular weight (HMW) DNA extraction developed at the Wellcome Sanger Institute (WSI) Tree of Life Core Laboratory are available on protocols.io ( Howard *et al.*, 2025). The ilKreTrap1 sample was weighed and triaged to determine the appropriate extraction protocol. Tissue from the thorax was homogenised by powermashing using a PowerMasher II tissue disruptor.

HMW DNA was extracted in the WSI Scientific Operations core using the Automated MagAttract v2 protocol. DNA was sheared into an average fragment size of 12–20 kb following the Megaruptor®3 for LI PacBio protocol. Sheared DNA was purified by automated SPRI (solid-phase reversible immobilisation). The concentration of the sheared and purified DNA was assessed using a Nanodrop spectrophotometer and Qubit Fluorometer using the Qubit dsDNA High Sensitivity Assay kit. Fragment size distribution was evaluated by running the sample on the FemtoPulse system. For this sample, the final post-shearing DNA had a Qubit concentration of 42.4 ng/μL and a yield of 1 992.80 ng, with a fragment size of 15.1 kb. The 260/280 spectrophotometric ratio was 1.84, and the 260/230 ratio was 2.58.

### PacBio HiFi library preparation and sequencing

Library preparation and sequencing were performed at the WSI Scientific Operations core. Libraries were prepared using the SMRTbell Prep Kit 3.0 Pacific Biosciences, California, USA), following the manufacturer’s instructions. The kit includes reagents for end repair/A-tailing, adapter ligation, post-ligation SMRTbell bead clean-up, and nuclease treatment. Size selection and clean-up were performed using diluted AMPure PB beads (Pacific Biosciences). DNA concentration was quantified using a Qubit Fluorometer v4.0 (ThermoFisher Scientific) and the Qubit 1X dsDNA HS assay kit. Final library fragment size was assessed with the Agilent Femto Pulse Automated Pulsed Field CE Instrument (Agilent Technologies) using the gDNA 55 kb BAC analysis kit.

The sample was sequenced on a Revio instrument (Pacific Biosciences). The prepared library was normalised to 2 nM, and 15 μL was used for making complexes. Primers were annealed and polymerases bound to generate circularised complexes, following the manufacturer’s instructions. Complexes were purified using 1.2X SMRTbell beads, then diluted to the Revio loading concentration (200–300 pM) and spiked with a Revio sequencing internal control. The sample was sequenced on a Revio 25M SMRT cell. The SMRT Link software (Pacific Biosciences), a web-based workflow manager, was used to configure and monitor the run and to carry out primary and secondary data analysis.

Specimen details, sequencing platforms, and data yields are summarised in Table 1.

**Table 1.** Specimen and sequencing data for BioProject.

Platform	PacBio HiFi	Hi-C
**ToLID**	ilKreTrap1	ilKreTrap1
**Specimen ID**	SAN28000118	SAN28000118
**BioSample (source individual)**	SAMEA115109935	SAMEA115109935
**BioSample (tissue)**	SAMEA115109954	SAMEA115109955
**Tissue**	thorax	head
**Sequencing platform and model**	Revio	Illumina NovaSeq X
**Run accessions**	ERR13605506	ERR13602169
**Read count total**	3.71 million	888.79 million
**Base count total**	41.42 Gb	134.21 Gb

### Hi-C

***Sample preparation and crosslinking***

The Hi-C sample was prepared from 20–50 mg of frozen head tissue of the ilKreTrap1 sample using the Arima-HiC v2 kit (Arima Genomics). Following the manufacturer’s instructions, tissue was fixed and DNA crosslinked using TC buffer to a final formaldehyde concentration of 2%. The tissue was homogenised using the Diagnocine Power Masher-II. Crosslinked DNA was digested with a restriction enzyme master mix, biotinylated, and ligated. Clean-up was performed with SPRISelect beads before library preparation. DNA concentration was measured with the Qubit Fluorometer (Thermo Fisher Scientific) and Qubit HS Assay Kit. The biotinylation percentage was estimated using the Arima-HiC v2 QC beads.

***Hi-C library preparation and sequencing***

Biotinylated DNA constructs were fragmented using a Covaris E220 sonicator and size selected to 400–600 bp using SPRISelect beads. DNA was enriched with Arima-HiC v2 kit Enrichment beads. End repair, A-tailing, and adapter ligation were carried out with the NEBNext Ultra II DNA Library Prep Kit (New England Biolabs), following a modified protocol where library preparation occurs while DNA remains bound to the Enrichment beads. Library amplification was performed using KAPA HiFi HotStart mix and a custom Unique Dual Index (UDI) barcode set (Integrated DNA Technologies). Depending on sample concentration and biotinylation percentage determined at the crosslinking stage, libraries were amplified with 10–16 PCR cycles. Post-PCR clean-up was performed with SPRISelect beads. Libraries were quantified using the AccuClear Ultra High Sensitivity dsDNA Standards Assay Kit (Biotium) and a FLUOstar Omega plate reader (BMG Labtech).

Prior to sequencing, libraries were normalised to 10 ng/μL. Normalised libraries were quantified again and equimolar and/or weighted 2.8 nM pools. Pool concentrations were checked using the Agilent 4200 TapeStation (Agilent) with High Sensitivity D500 reagents before sequencing. Sequencing was performed using paired-end 150 bp reads on the Illumina NovaSeq X.

Specimen details, sequencing platforms, and data yields are summarised in Table 1.

### Genome assembly

Prior to assembly of the PacBio HiFi reads, a database of *k*-mer counts ( *k* = 31) was generated from the filtered reads using FastK. GenomeScope2 ( Ranallo-Benavidez *et al.*, 2020) was used to analyse the *k*-mer frequency distributions, providing estimates of genome size, heterozygosity, and repeat content.

The HiFi reads were assembled using Hifiasm in Hi-C phasing mode ( Cheng *et al.*, 2021; Cheng *et al.*, 2022), producing two haplotypes. Hi-C reads ( Rao *et al.*, 2014) were mapped to the primary contigs using bwa-mem2 ( Vasimuddin *et al.*, 2019). Contigs were further scaffolded with Hi-C data in YaHS ( Zhou *et al.*, 2023), using the --break option for handling potential misassemblies. The scaffolded assemblies were evaluated using Gfastats ( Formenti *et al.*, 2022), BUSCO ( Manni *et al.*, 2021) and MERQURY.FK ( Rhie *et al.*, 2020).

The mitochondrial genome was assembled using MitoHiFi ( Uliano-Silva *et al.*, 2023), which runs MitoFinder ( Allio *et al.*, 2020) and uses these annotations to select the final mitochondrial contig and to ensure the general quality of the sequence.

### Assembly curation

The assembly was decontaminated using the Assembly Screen for Cobionts and Contaminants ( ASCC) pipeline. TreeVal was used to generate the flat files and maps for use in curation. Manual curation was conducted primarily in PretextView and HiGlass ( Kerpedjiev *et al.*, 2018). Scaffolds were visually inspected and corrected as described by Howe *et al*. (2021). Manual corrections included 13 breaks and 50 joins. The curation process is documented at https://gitlab.com/wtsi-grit/rapid-curation.

### Assembly quality assessment

Chromosomal painting was performed using lep_busco_painter using Merian elements, which represent the 32 ancestral linkage groups in Lepidoptera ( Wright *et al.*, 2024). Painting was based on gene locations from the lepidoptera_odb10 BUSCO analysis and chromosome lengths from the genome index produced using SAMtools faidx ( Danecek *et al.*, 2021). Each complete BUSCO (including both single-copy and duplicated BUSCOs) was assigned to a Merian element using a reference database, and coloured positions were plotted along chromosomes drawn to scale.

The Merqury.FK tool ( Rhie *et al.*, 2020), run in a Singularity container ( Kurtzer *et al.*, 2017), was used to evaluate *k*-mer completeness and assembly quality for both haplotypes using the *k*-mer databases ( *k* = 31) computed prior to genome assembly. The analysis outputs included assembly QV scores and completeness statistics.

The genome was analysed using the BlobToolKit pipeline, a Nextflow implementation of the earlier Snakemake BlobToolKit pipeline ( Challis *et al.*, 2020). The pipeline aligns PacBio reads using minimap2 ( Li, 2018) and SAMtools ( Danecek *et al.*, 2021) to generate coverage tracks. Simultaneously, it queries the GoaT database ( Challis *et al.*, 2023) to identify relevant BUSCO lineages and runs BUSCO ( Manni *et al.*, 2021). For the three domain-level BUSCO lineages, BUSCO genes are aligned to the UniProt Reference Proteomes database ( Bateman *et al.*, 2023) using DIAMOND blastp ( Buchfink *et al.*, 2021). The genome is divided into chunks based on the density of BUSCO genes from the closest taxonomic lineage, and each chunk is aligned to the UniProt Reference Proteomes database with DIAMOND blastx. Sequences without hits are chunked using seqtk and aligned to the NT database with blastn ( Altschul *et al.*, 1990). The BlobToolKit suite consolidates all outputs into a blobdir for visualisation. The BlobToolKit pipeline was developed using nf-core tooling ( Ewels *et al.*, 2020) and MultiQC ( Ewels *et al.*, 2016), with package management via Conda and Bioconda ( Grüning *et al.*, 2018), and containerisation through Docker ( Merkel, 2014) and Singularity ( Kurtzer *et al.*, 2017).

## Genome sequence report

### Sequence data

The genome of a specimen of *Kretania trappi* was sequenced using Pacific Biosciences single-molecule HiFi long reads, generating 41.42 Gb (gigabases) from 3.71 million reads, which were used to assemble the genome. GenomeScope2.0 analysis estimated the haploid genome size at 623.81 Mb, with a heterozygosity of 0.75% and repeat content of 51.78%. These estimates guided expectations for the assembly. Based on the estimated genome size, the sequencing data provided approximately 64× coverage. Hi-C sequencing produced 134.21 Gb from 888.79 million reads, which were used to scaffold the assembly.

Table 1 summarises the specimen and sequencing details.

### Assembly statistics

The genome was assembled into two haplotypes using Hi-C phasing. Haplotype 1 was curated to chromosome level, while haplotype 2 was assembled to scaffold level. The final assembly has a total length of 683.81 Mb in 121 scaffolds, with 435 gaps, and a scaffold N50 of 32.72 Mb ( Table 2).

**Table 2.** Genome assembly statistics.

**Assembly name**	ilKreTrap1.hap1.1	ilKreTrap1.hap2.1
**Assembly accession**	GCA_964264435.1	GCA_964264395.1
**Assembly level**	chromosome	scaffold
**Span (Mb)**	683.81	597.58
**Number of chromosomes**	20	N/A
**Number of contigs**	556	449
**Contig N50**	2.69 Mb	2.69 Mb
**Number of scaffolds**	121	99
**Scaffold N50**	32.72 Mb	32.12 Mb
**Longest scaffold length (Mb)**	59.93	N/A
**Sex chromosomes**	W and Z	N/A
**Organelles**	Mitochondrial genome: 15.35 kb	N/A

**Figure 2.**
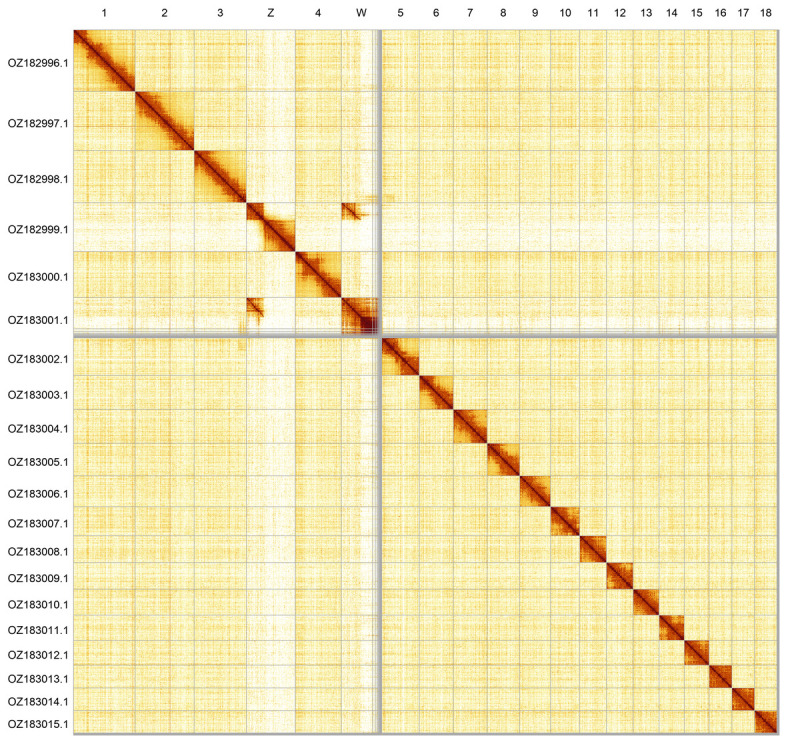
Hi-C contact map of the *Kretania trappi* genome assembly. Assembled chromosomes are shown in order of size and labelled along the axes. The plot was generated using PretextSnapshot.

**Figure 3.**
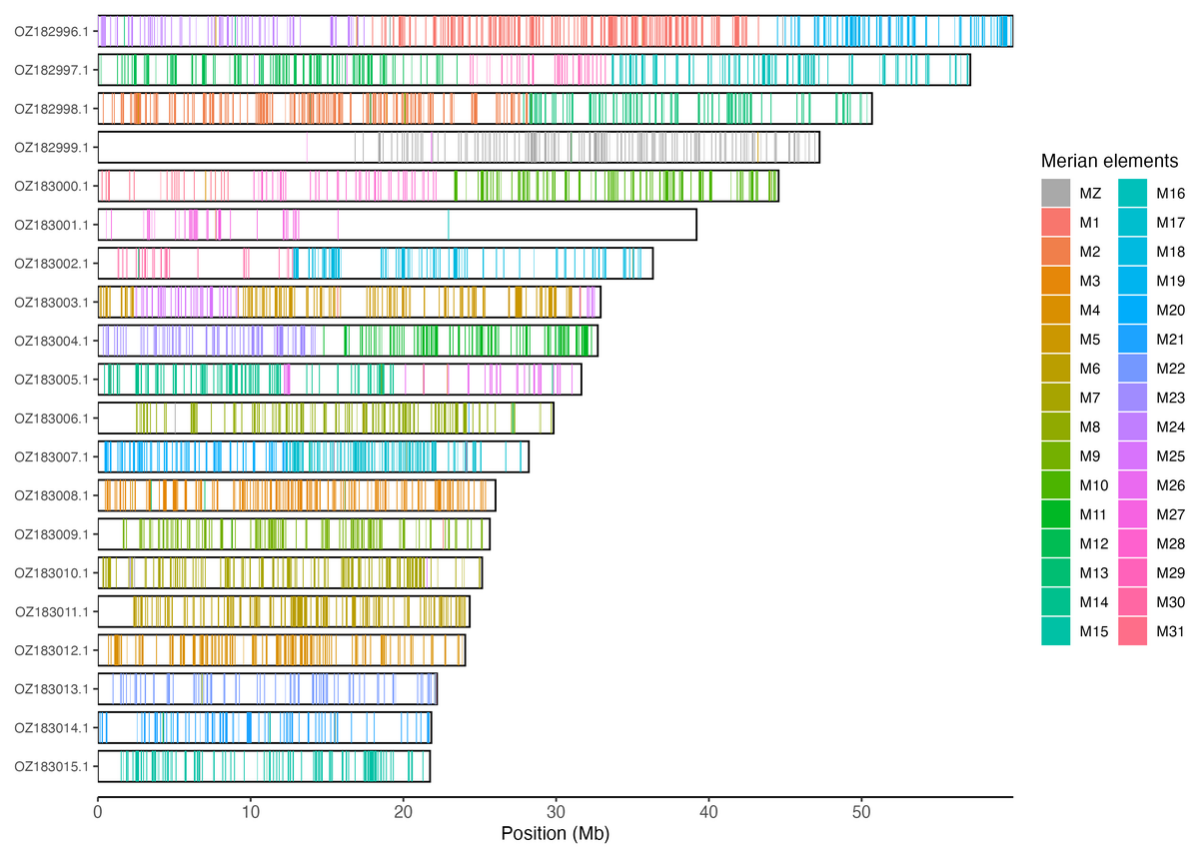
Merian elements painted across chromosomes in the ilKreTrap1.hap1.1 assembly of *Kretania trappi*. Chromosomes are drawn to scale, with the positions of orthologues shown as coloured bars. Each orthologue is coloured by the Merian element that it belongs to. All orthologues which could be assigned to Merian elements are shown.

Most of the assembly sequence (98.36%) was assigned to 20 chromosomal-level scaffolds, representing 18 autosomes and the W and Z sex chromosomes. These chromosome-level scaffolds, confirmed by Hi-C data, are named according to size ( Figure 2; Table 3). Chromosome painting with Merian elements illustrates the distribution of orthologues along chromosomes and highlights patterns of chromosomal evolution relative to Lepidopteran ancestral linkage groups ( Figure 3).

**Table 3.** Chromosomal pseudomolecules in the haplotype 1 genome assembly of *Kretania trappi* ilKreTrap1.

INSDC accession	Molecule	Length (Mb)	GC%	Assigned Merian elements
OZ182996.1	1	59.93	37	M1;M19;M24
OZ182997.1	2	57.11	37	M12;M16;M29
OZ182998.1	3	50.68	37	M13;M2
OZ183000.1	4	44.56	37	M10;M28;M31
OZ183002.1	5	36.34	37.50	M18;M30
OZ183003.1	6	32.91	37	M25;M5
OZ183004.1	7	32.72	37	M11;M23
OZ183005.1	8	31.65	37	M14;M26
OZ183006.1	9	29.83	37	M8
OZ183007.1	10	28.21	37	M17;M20
OZ183008.1	11	26.03	37.50	M3
OZ183009.1	12	25.66	36.50	M9
OZ183010.1	13	25.15	37	M7
OZ183011.1	14	24.34	37.50	M6
OZ183012.1	15	24.05	37	M4
OZ183013.1	16	22.20	37	M22
OZ183014.1	17	21.83	37	M21
OZ183015.1	18	21.74	37	M15
OZ183001.1	W	39.19	37.50	M27
OZ182999.1	Z	47.24	37	MZ

The mitochondrial genome was also assembled. This sequence is included as a contig in the multifasta file of the genome submission and as a standalone record.

### Assembly quality metrics

**Figure 4.**
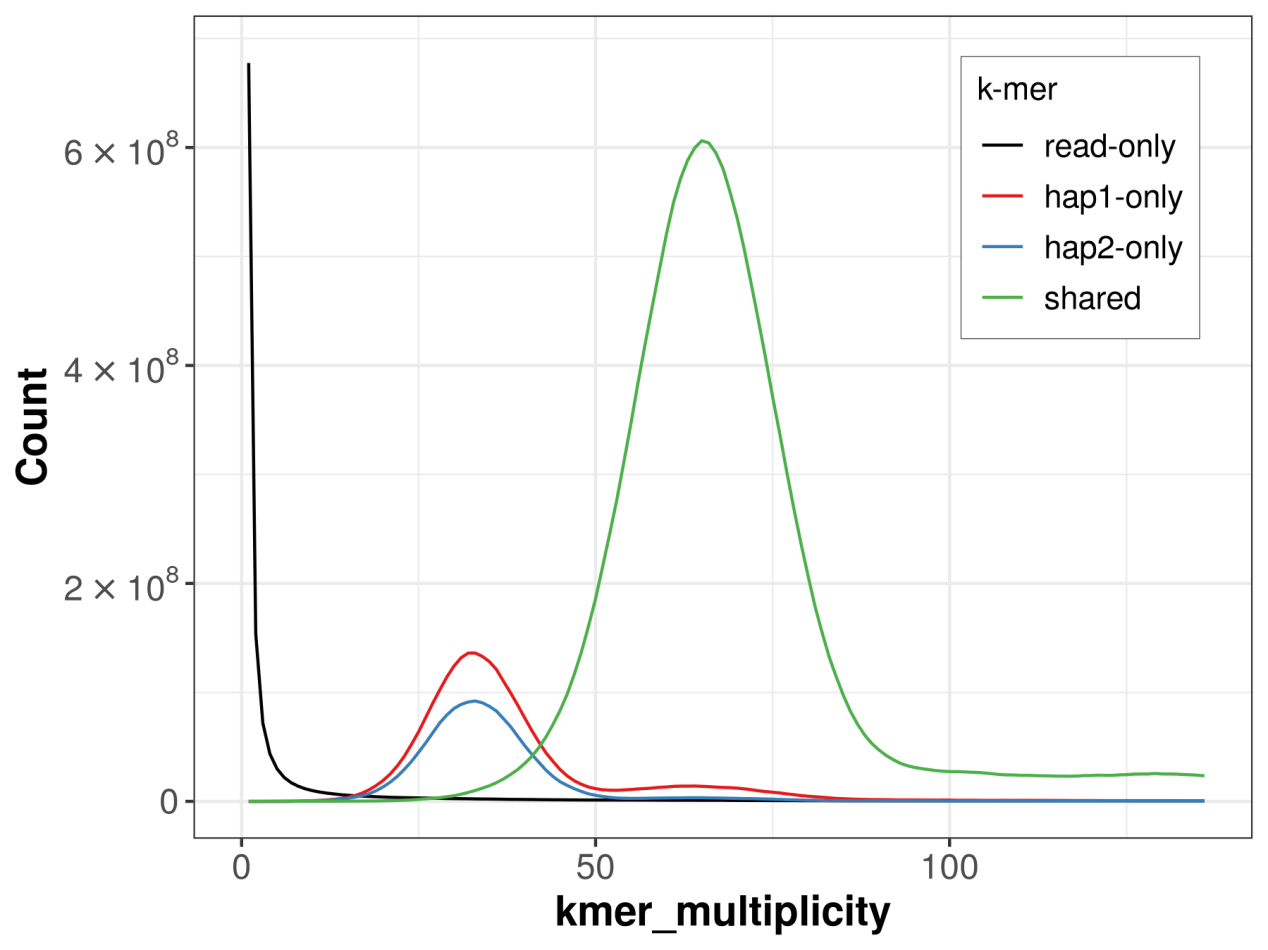
Evaluation of *k*-mer completeness using MerquryFK. This plot illustrates the recovery of *k*-mers from the original read data in the final assemblies. The horizontal axis represents *k*-mer multiplicity, and the vertical axis shows the number of *k*-mers. The black curve represents *k*-mers that appear in the reads but are not assembled. The green curve (the homozygous peak) corresponds to *k*-mers shared by both haplotypes and the red and blue curves (the heterozygous peaks) show *k*-mers found only in one of the haplotypes.

**Figure 5.**
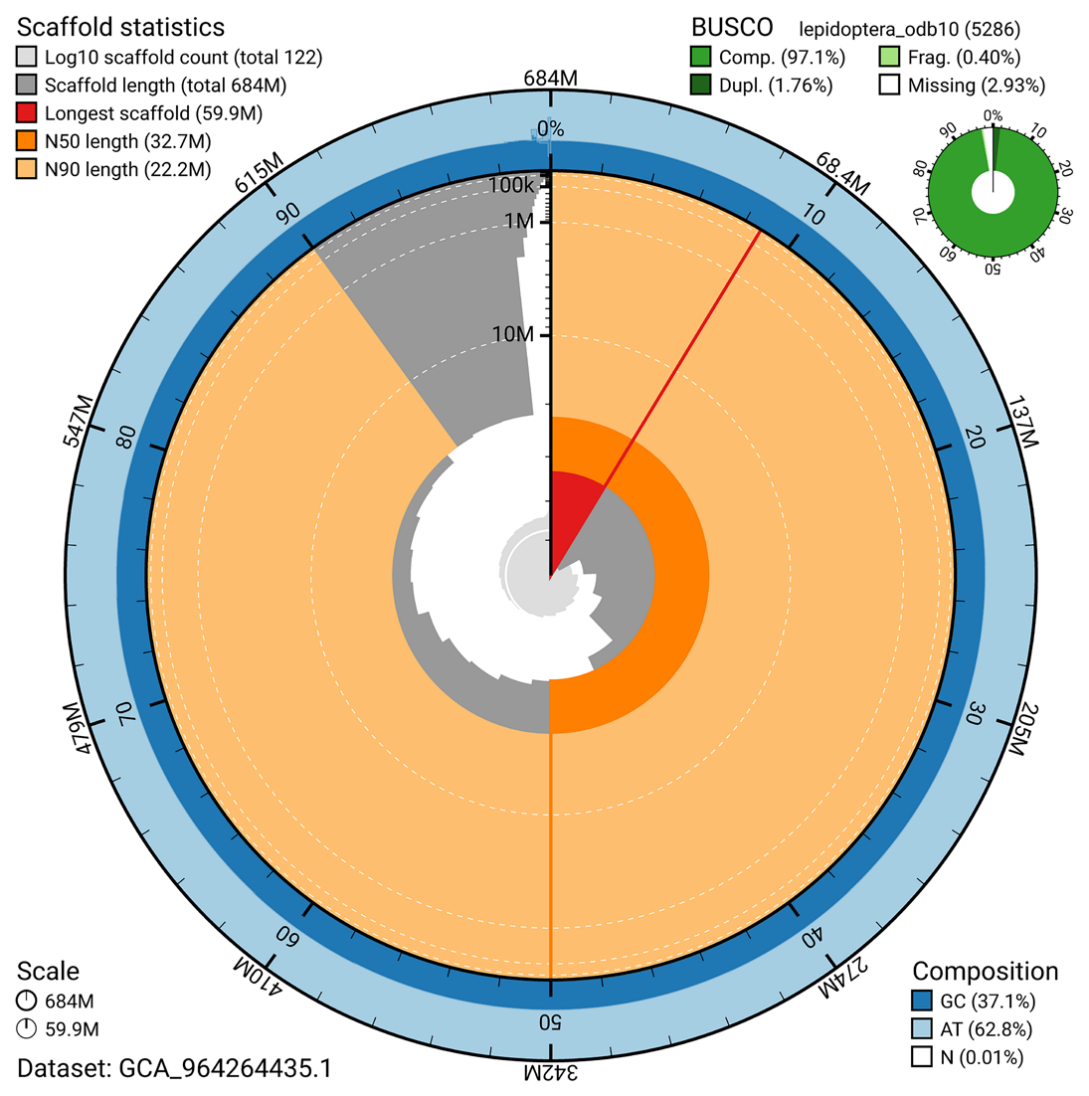
Assembly metrics for ilKreTrap1.hap1.1. The BlobToolKit snail plot provides an overview of assembly metrics and BUSCO gene completeness. The circumference represents the length of the whole genome sequence, and the main plot is divided into 1,000 bins around the circumference. The outermost blue tracks display the distribution of GC, AT, and N percentages across the bins. Scaffolds are arranged clockwise from longest to shortest and are depicted in dark grey. The longest scaffold is indicated by the red arc, and the deeper orange and pale orange arcs represent the N50 and N90 lengths. A light grey spiral at the centre shows the cumulative scaffold count on a logarithmic scale. A summary of complete, fragmented, duplicated, and missing BUSCO genes in the set is presented at the top right. An interactive version of this figure can be accessed on the BlobToolKit viewer.

**Figure 6.**
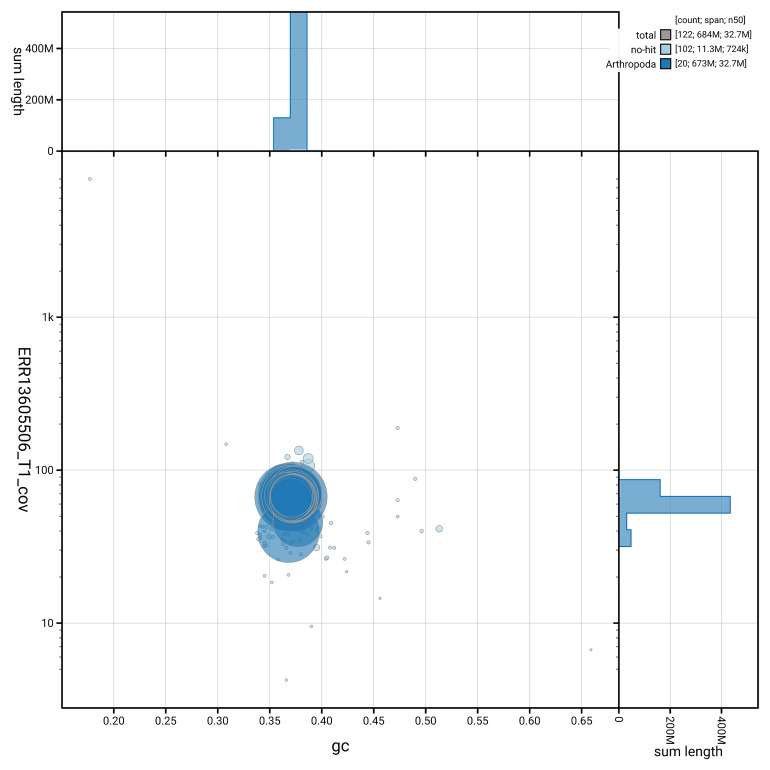
BlobToolKit GC-coverage plot for ilKreTrap1.hap1.1. Blob plot showing sequence coverage (vertical axis) and GC content (horizontal axis). The circles represent scaffolds, with the size proportional to scaffold length and the colour representing phylum membership. The histograms along the axes display the total length of sequences distributed across different levels of coverage and GC content. An interactive version of this figure is available on the BlobToolKit viewer.

For haplotype 1, the estimated QV is 61.9, and for haplotype 2, 62.6. When the two haplotypes are combined, the assembly achieves an estimated QV of 62.2. The *k*-mer completeness is 86.46% for haplotype 1, 79.73% for haplotype 2, and 98.85% for the combined haplotypes ( Figure 4). BUSCO analysis using the lepidoptera_odb10 reference set ( *n* = 5 286) ( Kriventseva *et al.*, 2019) identified 97.1% of the expected gene set (single = 95.3%, duplicated = 1.8%) for haplotype 1. The snail plot in Figure 5 summarises the scaffold length distribution and other assembly statistics for haplotype 1. The blob plot in Figure 6 shows the distribution of scaffolds by GC proportion and coverage for haplotype 1.

Table 4 lists the assembly metric benchmarks adapted from Rhie *et al.* (2021) the Earth BioGenome Project Report on Assembly Standards September 2024. The EBP metric, calculated for the haplotype 1, is **6.C.Q61**, meeting the recommended reference standard.

**Table 4.** Earth Biogenome Project summary metrics for the *Kretania trappi* assembly.

Measure	Value	Benchmark
EBP summary (haplotype 1)	6.7.Q61	6.C.Q40
Contig N50 length	2.69 Mb	≥ 1 Mb
Scaffold N50 length	32.72 Mb	= chromosome N50
Consensus quality (QV)	Haplotype 1: 61.9; haplotype 2: 62.6; combined: 62.2	≥ 40
*k*-mer completeness	Haplotype 1: 86.46%; Haplotype 2: 79.73%; combined: 98.85%	≥ 95%
BUSCO	C:97.1%[S:95.3%‚D:1.8%]‚ F:0.4%‚M:2.5%‚n:5 286	S > 90%; D < 5%
Percentage of assembly assigned to chromosomes	98.36%	≥ 90%

### Wellcome Sanger Institute – Legal and Governance

The materials that have contributed to this genome note have been supplied by a Tree of Life collaborator. The Wellcome Sanger Institute employs a process whereby due diligence is carried out proportionate to the nature of the materials themselves, and the circumstances under which they have been/are to be collected and provided for use. The purpose of this is to address and mitigate any potential legal and/or ethical implications of receipt and use of the materials as part of the research project, and to ensure that in doing so, we align with best practice wherever possible. The overarching areas of consideration are:

- Ethical review of provenance and sourcing of the material
- Legality of collection, transfer and use (national and international).

Each transfer of samples is undertaken according to a Research Collaboration Agreement or Material Transfer Agreement entered into by the Tree of Life collaborator, Genome Research Limited (operating as the Wellcome Sanger Institute), and in some circumstances, other Tree of Life collaborators.

## Data Availability

European Nucleotide Archive: Kretania trappi (Alpine zephyr blue). Accession number PRJEB79186. The genome sequence is released openly for reuse. The *Kretania trappi* genome sequencing initiative is part of the Sanger Institute Tree of Life Programme (PRJEB43745) and Project Psyche (PRJEB71705). All raw sequence data and the assembly have been deposited in INSDC databases. The genome will be annotated using available RNA-Seq data and presented through Ensembl at the European Bioinformatics Institute. Raw data and assembly accession identifiers are reported in Table 1 and Table 2. Pipelines used for genome assembly at the WSI Tree of Life are available at https://pipelines.tol.sanger.ac.uk/pipelines. Table 5 lists software versions used in this study.
