# Supplementary material for: The genome sequence of the Alpine Zephyr Blue, Kretania trappi (Verity, 1927) (Lepidoptera: Lycaenidae)
Source: Wellcome Open Res. 2025 Aug 11;10:432. [Version 1] doi: 10.12688/wellcomeopenres.24703.1 (PMC12464535; doi:10.12688/wellcomeopenres.24703.1)
Supplement: Peer review history

# Reviewer report: Reviewer response for version 1

**DOI:** 10.21956/wellcomeopenres.27219.r130691

**Published:** 2025-09-25

**Authors:** Aleksandra Gwiazdowska

**Affiliations:** 1 Museum and Institute of Zoology Polish Academy of Sciences (Ringgold ID: 86918), Warsaw, Masovian Voivodeship, Poland; 2 Polish Academy of Sciences, Twarda, Poland

The authors described the reference genome of Alpine Zephyr Blue ( *Kretania trappi*). The introduction has the most important information about the species. The photograph of the *Kretania trappi *voucher is of very good quality and consists of a scale. The methods are described in great detail, and each part has a link to see the details on the web side. The pipeline is available online, and the reference genome has an accession number.

 In summary, the results and methods are available and reproducible. The reference genome will help for future studies, for example, taxonomic and conservation genomics studies.

Are sufficient details of methods and materials provided to allow replication by others?

Yes

Is the rationale for creating the dataset(s) clearly described?

Yes

Are the datasets clearly presented in a useable and accessible format?

Yes

Are the protocols appropriate and is the work technically sound?

Yes

Reviewer Expertise:

Population Genetics, Conservation Genetics

I confirm that I have read this submission and believe that I have an appropriate level of expertise to confirm that it is of an acceptable scientific standard.

# Reviewer report: Reviewer response for version 1

**DOI:** 10.21956/wellcomeopenres.27219.r131571

**Published:** 2025-09-23

**Authors:** Vlad Eugen Dinca

**Affiliations:** 1 National History Museum Grigore Antipa (Ringgold ID: 277205), Bucharest, Bucharest, Romania; 2 University of Oulu Centre of Excellence in Cell-Extracellular Matrix Research, Oulu, Northern Ostrobothnia, Finland

In this data note, the authors have successfully generated a chromosome-level genome assembly of a female *Kretania trappi* (Lepidoptera: Lycaenidae). The mitochondrial genome has been assembled as well.

 Although I am not an expert in several of the methodological approaches used, as far as I can tell, the manuscript is technically sound and uses methodologies and pipelines that are fairly well-established through use by the Sanger Institute.

 The genome appears to be of high quality and scaffolded into 20 chromosomal pseudomolecules (18 autosomes, as well as the W and Z sex chromosomes).

 Reference genomes such as this one represent a valuable resource for the scientific community and each new addition provides new opportunities for research. This genome is also a notable step forward towards resolving the *pylaon* species complex, which has been subject to considerable debate.

 A few additional comments are included below.

 A checklist of W Palearctic butterflies was published  by Dapporto et al. (2022) [Reference 1]. *Kretania trappi* was also listed as a species there.

 I would replace "Rumania" with "Romania".

 Please replace " *Plebeius*" with " *Plebejus*".

 The intraspecific divergence based on COI is not pronounced. Dapporto et al. (2022) indicate a max. p-dist. of 0.3%. Thus, I suggest rephrasing the last sentence of the Background. It may be mentioned that the Val Venosta populations seem to display some degree of differentiation from other populations.

 Figure 1: If possible, for the voucher, it would also be good to include an image of the ventral side of the wings.

Are sufficient details of methods and materials provided to allow replication by others?

Yes

Is the rationale for creating the dataset(s) clearly described?

Yes

Are the datasets clearly presented in a useable and accessible format?

Yes

Are the protocols appropriate and is the work technically sound?

Yes

Reviewer Expertise:

Lepidoptera taxonomy, phylogeography, conservation

I confirm that I have read this submission and believe that I have an appropriate level of expertise to confirm that it is of an acceptable scientific standard.

## References

[1] : The atlas of mitochondrial genetic diversity for Western Palaearctic butterflies. *Global Ecology and Biogeography* .2022;31(11) : 10.1111/geb.13579 2184-2190 10.1111/geb.13579
